# Transplant Trial Watch

**DOI:** 10.3389/ti.2023.11920

**Published:** 2023-09-27

**Authors:** John Matthew O’Callaghan, Simon Knight

**Affiliations:** ^1^ University Hospitals Coventry and Warwickshire NHS Trust, Coventry, United Kingdom; ^2^ Peter Morris Centre for Evidence in Transplantation, University of Oxford, Oxford, United Kingdom; ^3^ Nuffield Department of Surgery, University of Oxford, Oxford, United Kingdom

**Keywords:** randomised controlled trial, liver transplantation, heart transplantation, donation after brain death, donation after circulatory death (DCD)

To keep the transplantation community informed about recently published level 1 evidence in organ transplantation ESOT and the Centre for Evidence in Transplantation have developed the Transplant Trial Watch. The Transplant Trial Watch is a monthly overview of 10 new randomised controlled trials (RCTs) and systematic reviews. This page of Transplant International offers commentaries on methodological issues and clinical implications on two articles of particular interest from the CET Transplant Trial Watch monthly selection. For all high quality evidence in solid organ transplantation, visit the Transplant Library: www.transplantlibrary.com.

RANDOMISED CONTROLLED TRIAL 1 Transplantation Outcomes with Donor Hearts after Circulatory Death.

*by Schroder, J. N., et al. New England Journal of Medicine 2023; 388(23): 2121–2131*.

## Aims

The aim of this study was to investigate posttransplant outcomes of hearts obtained from donation after circulatory death (DCD) versus donation after brain death (DBD) donors.

## Interventions

Participants were randomised to receive a heart from either a DCD or DBD donor.

## Participants

297 adult candidates for heart transplantation were randomised, out of which 180 underwent transplantation.

## Outcomes

The primary efficacy outcome was patient survival adjusted for prespecified donor and recipient risk factors. The secondary efficacy outcome was the donor-heart utilization rate.

## Follow-Up

1 year posttransplantation.

## CET Conclusion

This multicentre study randomised patients on the heart transplant waiting list to waiting for a standard, DBD organ; or to a DCD organ (assessed via *ex-vivo* perfusion) or DBD organ, whichever came first. 297 wait-listed patients were randomised, of whom 180 were transplanted in the study – 90 with DBD organs, and 90 with DCD organs. At 6 months post-transplant, there was no difference in risk-adjusted survival or other clinical outcomes between the two groups. This is a very well-designed study. Studies that alter with organ allocation are challenging as they must not disadvantage patients by reducing the chances of an organ offer. By allowing patients in the DCD arm to receive a DBD organ if allocated, the investigators overcome this. At least in the short term, outcomes from DCD hearts assessed *ex-vivo* appear equivalent, and have the potential to increase the rate of transplantation – within the study, 67% patients randomised to the DCD cohort were transplanted compared to 39% in the DBD cohort.

## Jadad Score

2.

## Data Analysis

Per protocol analysis.

## Allocation Concealment

No.

## Trial Registration

ClinicalTrials.gov—NCT03831048.

## Funding Source

Industry funded.

RANDOMISED CONTROLLED TRIAL 2 A randomized-controlled trial of ischemia-free liver transplantation for end-stage liver disease.

*by Guo, Z., et al. Journal of Hepatology 2023 [record in progress].*

## Aims

To compare outcomes in the novel technique of ischaemia-free liver transplantation (IFLT) to conventional liver transplantation (CLT).

## Interventions

The technique being tested is IFLT compared with CLT. IFLT is a complex technique in which during DBD donation the perfusion cannulas of a Liver Assist can be placed in the donor liver prior to cessation of donor circulation. The arterial canula placed via the splenic artery, portal vein via and vein graft and the outflow canula into the infra-hepatic cava. The perfusion can then seamlessly be transferred from donor circulation to NMP, the liver is then procured and continued NMP until implantation. The supra-hepatic caval (piggyback), portal vein and hepatic arterial anastomoses are then performed in the recipient while NMP continues, and once completed the NMP cannulas are removed, and hepatic perfusion transferred from NMP to recipient without interruption of perfusion.

## Participants

65 adult whole liver-only transplant recipients.

## Outcomes

The primary endpoint was early allograft dysfunction (EAD) within 7 days as defined by the Olthoff criteria. The secondary endpoints included primary non-function, post-reperfusion syndrome, biliary complications, post-reperfusion lactate, post-transplant LFTs, patient and graft survival at 1, 6, and 12 months, ITU stay and overall hospital stay.

## Follow-Up

12 months.

## CET Conclusion

This small unblinded randomised trial was conducted in a single high volume transplant centre in China by the group who have been pioneering the ischaemia-free liver transplant technique since its first publication in 2018. Images and videos of their technique have been included in their 3 publications on their reports and protocols. The IFLT cohort was *n* = 32 and the CLT *n* = 33, of these 2 (6%) in the IFLT experience EAD and 8 (24%) in the CLT (*p* = 0.044) which was the primary endpoint. In some of the secondary endpoints they found significant improvement with IFLT: peak ALT and ASK at 7 days, total bilirubin, post-op lactate positive perfusate microbial culture and non-anastomotic strictures at 12 months. When scrutinising these strictures, there were 2 in IFLT (one mild and one moderate) and 9 in CLT (five mild and four moderate) none of which required intervention. The marked reduction in post-reperfusion syndrome is important 3 (9%) in IFLT and 21 (64%) in CLT given the risk of post-reperfusion cardiac arrest. They found no significant differences in primary non-function, over-all hospital stay, anastomotic stenosis (though the rate was higher in IFLT) and, graft and patient survival. They present an impressive success given the complexity of the procedure, however this is its key limitation. Despite the improvement in EAD, strictures and post-reperfusion syndrome there was no measurable benefit in patient or graft survival within the first year and none of the strictures require intervention. It was done in a set of low risk DBD donors, a cohort in which similar benefits have been seen with NMP alone. There are technical limitations, it was performed with a liver assist device which is not transportable, thus donor and recipient must be in the same location. The technique is of interest and a great technical achievement, but a study of larger numbers with a wider range of DBD donors and longer-term follow-up is required.

## Jadad Score

3.

## Data Analysis

Modified intention-to-treat analysis.

## Allocation Concealment

Yes.

## Trial Registration

ChiCTR1900021158.

## Funding Source

Non-industry funded.

## Clinical Impact Summary

This is a very interesting randomised controlled trial in liver transplantation that has the potential to significantly change practice and improve transplant outcomes. 68 liver transplant recipients from donation after brain death were randomised to standard treatment or for an “Ischemia-Free Liver Transplant” (IFLT). The trial was conducted at a single hospital in China. The study was adequately randomised, but the clinical team could not be blinded to the intervention, understandably. For the intervention group, the Liver Assist device (Organ assist, Netherlands) was used to establish *in situ* normothermic perfusion. The liver was then procured and moved to the reservoir of the Liver Assist for *ex situ* normothermic machine perfusion and moved to the recipient locality for transplant. For the liver implantation to the recipient, the anastomoses of the inferior vena cava, portal vein, and hepatic artery were performed under continuous *in situ* normothermic machine perfusion. Machine perfusion was discontinued after the donor liver had been revascularized. Then the biliary tract was reconstructed.

There was therefore zero cold ischemic time for the IFLT group. Mean cold ischaemic time in the standard care group was approximately 7 h, and mean normothermic perfusion time in the IFLT group was approximately 7 h.

The primary outcome was Early Allograft Dysfunction (EAD) and this was significantly reduced by IFLT (6% versus 24%), as were peak ALT, AST and bilirubin levels. Post-reperfusion syndrome was dramatically reduced, from 64% to 9%. Non-anastomotic biliary strictures were also significantly reduced (8% versus 36%), although this was recorded as seen on protocol MRCP.

This clinical trial has shown a dramatic reduction in the ischemia reperfusion injury of transplant livers through the novel use of technology to remove the cold ischemic phase of the organ preservation period. The donor liver is kept warm and perfused all through the process of procurement from the donor body, preservation outside the body, and during the implant into the recipient up until the moment of reperfusion with the recipient’s blood. The technique clearly improved early transplant function. The reduction in non-anastomotic strictures was largely asymptomatic, so it remains to be seen if this technique can significantly reduce the risk of symptomatic strictures in higher risk livers.

